# Microplastics in marine mammals stranded around the British coast: ubiquitous but transitory?

**DOI:** 10.1038/s41598-018-37428-3

**Published:** 2019-01-31

**Authors:** S. E. Nelms, J. Barnett, A. Brownlow, N. J. Davison, R. Deaville, T. S. Galloway, P. K. Lindeque, D. Santillo, B. J. Godley

**Affiliations:** 1 Plymouth Marine Laboratory, Prospect Place, Plymouth, PL1 3DH UK; 2 Centre for Ecology and Conservation, University of Exeter, Cornwall, TR10 9EZ UK; 3 Environment and Sustainability Institute, University of Exeter, Cornwall, TR10 9EZ UK; 4 Scottish Marine Animal Stranding Scheme, SRUC Veterinary Services, Drummondhill, Inverness, IV2 4JZ UK; 5 Cetacean Strandings Investigation Programme, Institute of Zoology, Regent’s Park, London, NW1 4RY UK; 6 Biosciences, Geoffrey Pope Building, University of Exeter, Devon, EX4 4QD UK; 7 Greenpeace Research Laboratories, Innovation Centre Phase 2, University of Exeter, Devon, EX4 4RN UK

## Abstract

Plastic pollution represents a pervasive and increasing threat to marine ecosystems worldwide and there is a need to better understand the extent to which microplastics (<5 mm) are ingested by high trophic-level taxa, such as marine mammals. Here, we perform a comprehensive assessment by examining whole digestive tracts of 50 individuals from 10 species whilst operating strict contamination controls. Microplastics were ubiquitous with particles detected in every animal examined. The relatively low number per animal (mean = 5.5) suggests these particles are transitory. Stomachs, however, were found to contain a greater number than intestines, indicating a potential site of temporary retention. The majority of particles were fibres (84%) while the remaining 16% was fragments. Particles were mainly blue and black (42.5% and 26.4%) in colour and Nylon was the most prevalent (60%) polymer type. A possible relationship was found between the cause of death category and microplastic abundance, indicating that animals that died due to infectious diseases had a slightly higher number of particles than those that died of trauma and other drivers of mortality. It is not possible, however, to draw any firm conclusions on the potential biological significance of this observation and further research is required to better understand the potential chronic effects of microplastic exposure on animal health, particularly as marine mammals are widely considered important sentinels for the implications of pollution for the marine environment.

## Introduction

Marine mammals, such as whales, dolphins and seals, are often considered important indicators of marine ecosystem health, particularly in relation to pollution^1,2^. The high-trophic level status and long life-span of some species leaves them susceptible to bioaccumulation and biomagnification of aquatic chemical contaminants, which have been shown to cause population-level effects^3–5^. As a result of this and other anthropogenic stressors, many species of this taxonomic group are of conservation concern^6^. Ingestion of anthropogenic litter by marine mammals has been documented in a number of species (n = 123^7^), yet the number of studies (which use appropriate methods of extraction and contamination control) investigating the physical presence of microplastics (<5 mm in size) in the digestive tracts of cetaceans is extremely low (*n* = 4; totalling 57 animals of 8 species from Ireland, the Netherlands and Spain^8–11^; polymer information has been presented for two animals only^8,10^) and there are no studies whereby the digestive tracts of wild pinnipeds have been examined.

Microplastics in the marine environment originate from a variety of sources, including fragmentation of larger macro-plastic debris, pre-production pellets (nurdles) spilled during transportation and fabrication, outflow of wastewater containing microbeads from cosmetics and fibres from the washing of synthetic textiles, as well as road-run-off containing fragments of vehicle tyres and marking paint^12–17^. Their small size makes them highly bioavailable to ingestion by a wide variety of marine biota from zooplankton, such as copepods, other invertebrates (including shellfish), both juvenile and adult fish, seabirds and marine megafauna^8,10,18–24^.

Microplastics may be ingested directly through accidental consumption, for example as a result of indiscriminate feeding strategies, such as filter-feeding (e.g. mysticete whales^10^) or indirectly as a result of trophic transfer, whereby predators consume prey items contaminated with microplastics^19^, for example, during raptorial feeding (e.g. most seals and dolphins^25^). Though little is known about the extent to which trophic transfer occurs in the wild, the presence of microplastics in scats of captive grey seals (*Halichoerus grypus*) has been attributed to trophic transfer from the wild-caught mackerel (*Scomber scombrus*) they were fed upon^26^.

Due to the difficulties of investigating the occurrence and effects of microplastics in the field, many studies are limited to low-trophic level organisms in a laboratory setting. In such cases, ingestion of microplastics has been shown to cause a reduction in feeding and energy reserves as well as impacts on reproductive output and damage to brain and intestinal function in invertebrates and fish^18,27–30^. In addition, the hydrophobic properties of plastics means that organic chemical contaminants present within seawater, such as polychlorinated biphenyls (PCBs), have a tendency to adsorb to their surface^31^. These, and other chemicals added during production, such as plasticisers, can desorb into biological tissue if ingested and cause detrimental effects for organism health, such as oxidative and hepatic stress^32,33^.

In this study we sought to investigate the extent of microplastic ingestion in wild marine mammals by examining the digestive tracts of a large sample (*n* = 50) of individuals from 10 species (cetacean *n* = 43, 8 species; pinniped *n* = 7, 2 species) that stranded around the coast of Britain. We sought to not only determine the general abundance of microplastics ingested and polymers involved, but also to determine whether microplastics are egested or retained within the digestive tract.

## Results

### Microplastic abundance

**Figure 1:**
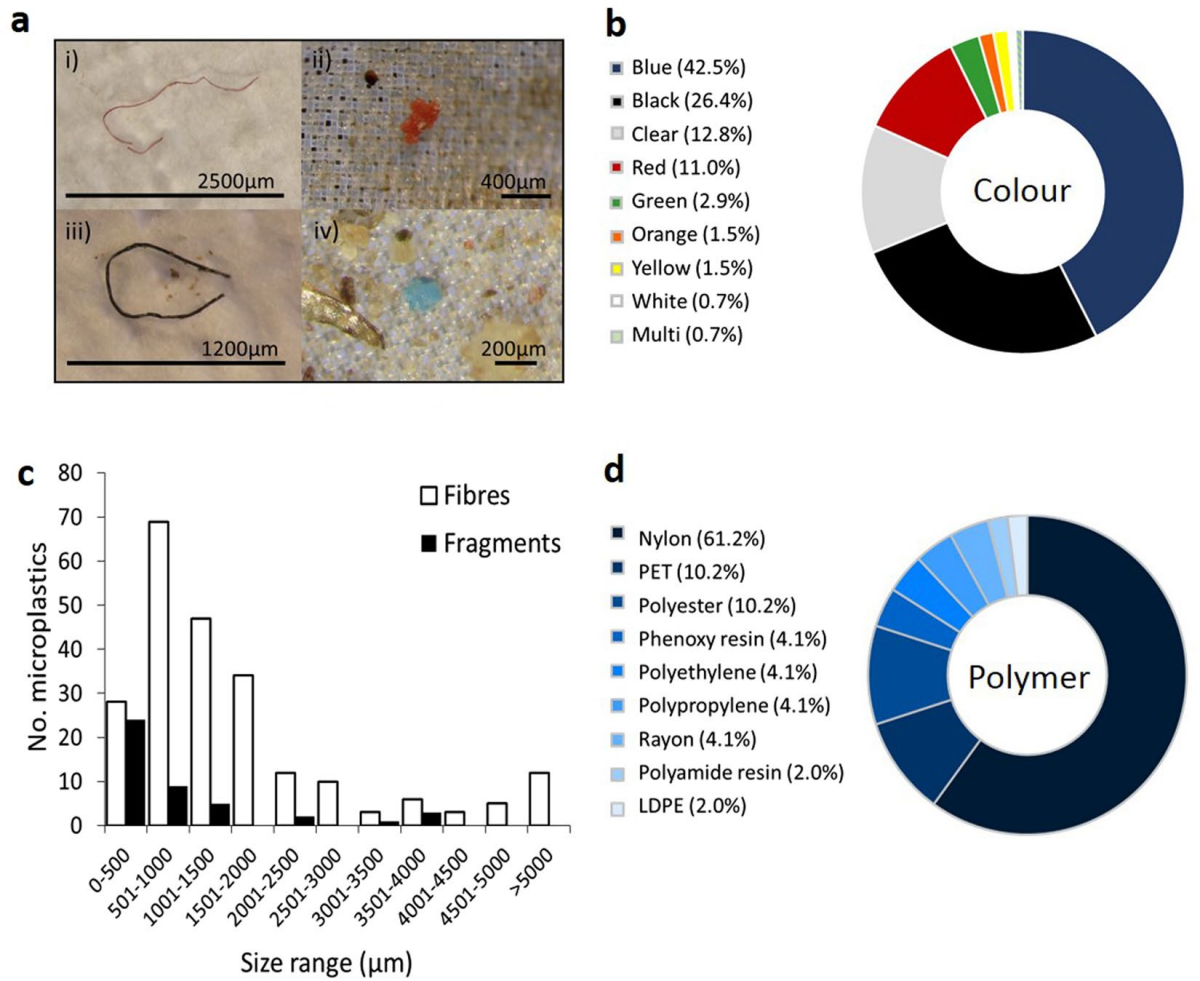
(**a**) Photographic examples of microplastics found in marine mammal digestive tracts (i) Nylon; (ii) Polyethylene; (iii) Polyethylene terephthalate (PET); (iv) Phenoxy resin (**b**) proportion of particle colours found in all animals (**c**) size ranges of particles found in all animals. Note: a small proportion of fibres were larger than 5 mm but were not macroscopically visible and are included here. (**d**) the proportion of polymer types found.

Every animal was found to contain at least one synthetic particle (See Fig. 1a for photographic examples). In total, 273 particles were detected and 261 of these were less than 5 mm in size (mean ± SD = 5.5 ± 2.7 particles per animal; range 1–12 particles). Only one animal was found to contain macroplastics; green netting in the forestomach of a juvenile short-beaked common dolphin (*Delphinus delphis*).

The majority of particles were fibres (84%; *n* = 229) while the remaining 16% (n = 44) was fragments. Particles were mainly blue and black (42.5% and 26.4%, respectively) followed by clear (12.8%), red (11%), green (2.9%), orange and yellow (both 1.5%) and white and multi-coloured (both 0.7%; Fig. 1b). Fibres ranged in size from 2 cm in length to 0.1 mm (100 µm) with a mean length of 2 mm ( ± 2.3 mm; Fig. 1c). Fragments were between 4 × 2 mm and 100 × 100 µm in size (mean length = 0.9 mm ± 1.1). All (100%; *n* = 50; one per animal) of the particles successfully tested using Fourier Transform Infrared (FTIR) spectroscopy were synthetic, with Nylon the most prevalent (60%; *n* = 30) followed by polyethylene terephthalate (PET) and polyester (all 10%; *n* = 5), phenoxy resin, polyethylene, polypropylene and rayon (all 4%; *n* = 2), polyamide resin and LDPE (both 2%; *n* = 1; Fig. 1d).

### Factors affecting microplastic abundance

**Figure 2:**
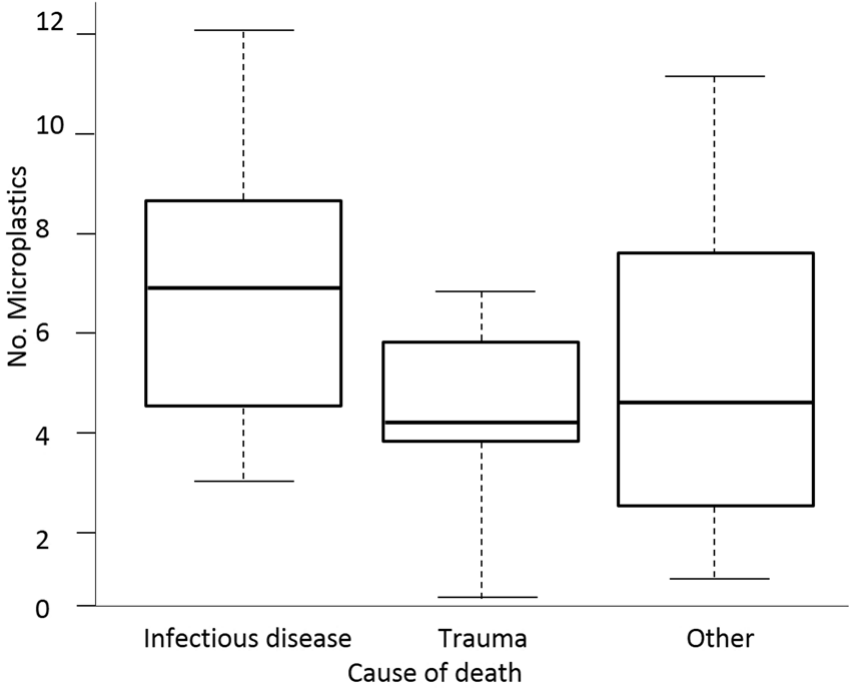
Box plot showing the number of microplastics in relation to cause of death category (infectious disease (7.0 ± 2.7), trauma (4.7 ± 2.1), other (4.6 ± 3.2)).

When we investigated factors that may affect microplastic burden (taxon, age-class, sex, length, cause of death), model simplification indicated that cause of death was the only significant predictor of microplastic abundance (*p* = 0.01; Supplementary Tables S1 and S2) and the mean number of microplastics was significantly different among the three cause of death categories (one-way ANOVA, F_2,47_ = 4.31, *p* < 0.05; Fig. 2). Animals presenting infectious diseases contained slightly higher mean ( ± SD) microplastics abundances (7.0 ± 2.7), followed by trauma (4.7 ± 2.1) and other (4.6 ± 3.2). This was also the case when we only analysed species (harbour porpoise and common dolphin) with sample size greater than 16 individuals. See Supplementary Tables S3 and S4 for further detail.

### Distribution of microplastics within the digestive tract

**Figure 3:**
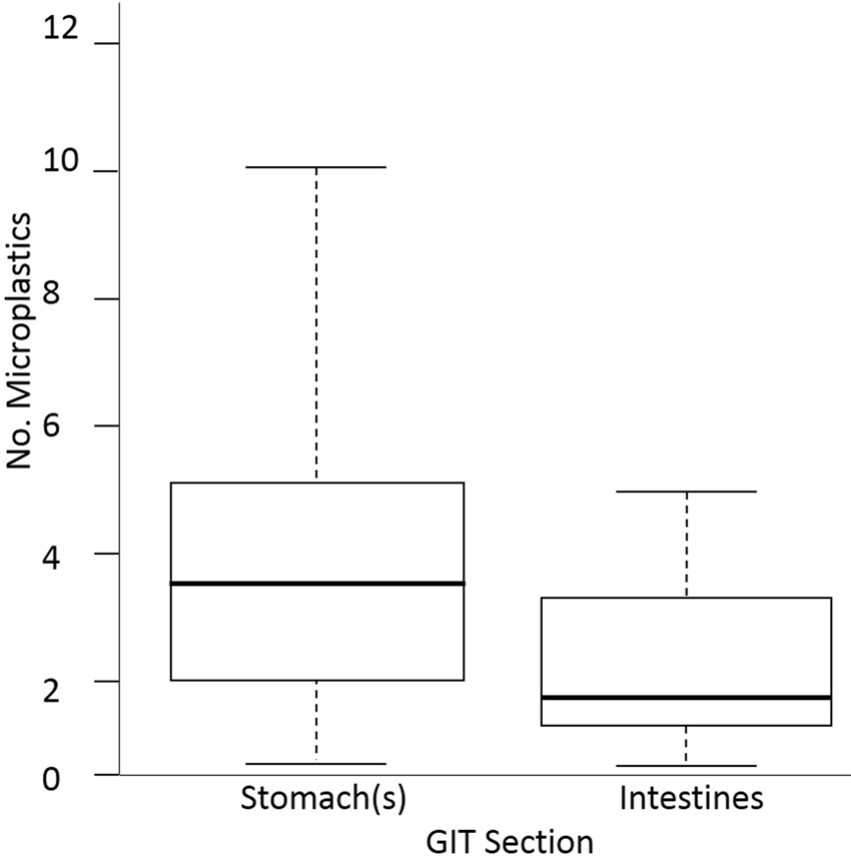
Box plot showing the number of microplastics detected in the gastro-intestinal tract (GIT) sections stomach(s) and intestines (mean ± SD = 3.8 particles ± 2.5 and 1.7 ± 1.4 respectively).

Of the GIT sections, stomach(s) showed a significantly higher abundance of microplastics (mean ± SD = 3.8 particles ± 2.5) than intestines (1.7 ± 1.4; one-way ANOVA, F_1,__98_ = 27.69, *p* < 0.001; Fig. 3.). There was no significant difference among the compartments of cetacean stomachs (fore, fundic and pyloric; ANOVA, F_2,__77_ = 0.6472, *p* = 0.5).

### Contamination

No particles matching the contamination controls were found in any of the samples and all procedural blanks were clear, demonstrating that the measures implemented to minimise contamination were 100% effective.

## Discussion

Our study is the first to assess the presence of microplastics in the digestive tracts of multiple individuals from a range of both cetacean and pinniped species. At least one microplastic, which was confirmed using FTIR, was discovered in every animal with an average of 5.5 particles per animal. There are few studies available for comparison but two studies examined the stomach contents of 35 common dolphins and digestive tracts of 21 cetaceans (of various species) and found a total of 411 and 598 small debris items respectively^9,11^. Neither study, however, presented FTIR data confirming polymer type. Sixteen confirmed microplastics were found in an unknown volume of gut content from a humpback whale (*Megaptera novaeangliae*)^10^.

All animals examined in the current study were raptorial feeders, using their jaws and teeth alone to catch prey^25^. As raptorial feeders expel seawater through their teeth so as not to ingest it, we presume they are less likely to consume microplastics directly and more likely to indirectly consume them through trophic transfer from contaminated prey^26^. However, given that approximately 11–30% of fish contain microplastics^21,34^ a greater number could perhaps be expected in the digestive tracts of marine mammals than demonstrated here. There are at least three possible explanations for the observed low abundances of microplastics. Firstly, microplastics are egested along with other dietary waste, such as fish bones, otoliths and squid beaks, as shown by their presence in seal scats and the intestines of both cetaceans and seals^8,26,35^. A feeding trial examining the passage time of prey in grey seals found the majority of otoliths were passed within four days of consumption and all polystyrene balls (3 mm) fed to the animals were recovered within six days, demonstrating that, although microplastics have a slower passage time, they are egested in the faeces^36,37^. Our finding of higher microplastic abundances in the stomach(s) than intestines, may explain this delay in passage time - the stomach(s) acts as an entrapment site within the digestive tract, partially retaining the microplastics. In addition to egestion, cetaceans, particularly odontocetes (toothed whales) are known to regurgitate foreign objects from the forestomach^38,39^, although very little information exists on the regurgitation rates of wild odontocetes^38^. Furthermore, a study on low trophic level organisms found microplastics transferred up food webs but were not present within predators after 10 days without exposure^40^. Secondly, the levels of microplastics in fish and other prey species may have been over-estimated due to poor contamination control in some studies^41^. For example, a study of North Sea fish found that 0.25% (1 out of 400) contained microplastics when, as undertaken in our study, strict quality assurance criteria were employed^41^. Lastly, the number of microplastics detected in this study possibly represents a proportion of what is actually present within the marine mammal GITs at the time of death as some may have been lost during the extraction process.

The majority of particles detected in our study were fibres, which corresponds with observations of environmental microplastic concentrations^42–44^ as well as those found in other studies on cetaceans, turtles and fish^8,9,21,34,45^. Similarly, blue and black, the most common colours detected in the marine mammal digestive tracts, frequently dominate composition of particles ingested by turtles, fish and zooplankton^21,23,24,45^. The mean length of fibres detected in the intestines of a True’s beaked whale was 2.16 mm which, again, corresponds closely with the mean length of fibres found in our study (2 mm)^8^. It is likely that, in our study and others, particles <500 µm in size are under-represented, due to detectability and size of mesh (35 µm) used for vacuum pumping.

In terms of polymer, previous studies found Nylon, polyethylene, polypropylene and polyethylene terephthalate which were also detected in our samples^8,10^.

Although a statistical relationship with a modest effect size was found between the cause-of-death category and microplastic abundance, it is not yet possible to draw firm conclusions on the potential biological significance of this observation. More research is required to better understand the potential chronic effects of microplastic exposure on marine mammal health. Sub-lethal effects, from the microplastics themselves or the chemical contaminants present on or within them are unlikely to be attributable to plastic ingestion at the low levels recorded here. It is not yet known to what extent microplastics act as a vector for transporting these toxincants from the aquatic environment into the tissues of marine mammals. It has been surmised that phthalates could act as a tracer for microplastic ingestion by Mediterranean fin whales (*Balaenoptera physalus*) because high concentrations of these plasticizers were detected in areas that corresponded with the spatial distribution of the whales^46^. To date, there is little empirical evidence to demonstrate a direct causal link between chemical contaminant load and microplastic ingestion in marine mammals. Potential health effects, such as depressed immune system function or increased vulnerability to diseases^47,48^, may not develop until after the microplastics have passed through the body. As a result, a causal relationship between microplastics and sub-lethal effects cannot be ruled out, especially where chronic exposure may lead to the bioaccumulation of toxicants. Additionally, inhalation of atmospheric microplastics^49^ by marine mammals may be a non-dietary source^9^, but the extent to which this occurs is currently unknown. Monitoring of at-sea atmospheric microplastic levels and examination of airways and lungs from stranded animals is needed.

In conclusion, we have shown that at least 10 of the 26 marine mammal species inhabiting or transiting through UK waters are exposed to microplastics through ingestion, though the potential for detrimental impacts is not known. Further examination of larger sample sizes, including investigation of animals of varying feeding strategies (e.g. lunge and suction feeders, such as baleen and beaked whales) in a greater variety of locations is required for comparison. Global hotspots for both large marine vertebrates and plastic pollution, such as the north-west Pacific Ocean^50,51^, may reveal clearer trends. In addition, investigation into the influence of oceanographic variables, such as currents, on both marine mammal strandings and marine litter may assist our understanding of their spatial relationship.

The methods employed in this study can be applied to a wide range of settings. Here, we were able to set baselines for geographical and temporal comparisons of microplastic ingestion within and across taxa. Exposure to microplastics is likely to be chronic, cumulative and persistent. Although the snapshot provided by this study cannot yet assess this risk, it does suggest that impacts of microplastic ingestion could manifest in these apex species, and hence further work is needed.

## Methods

### Sample collection

**Figure 4:**
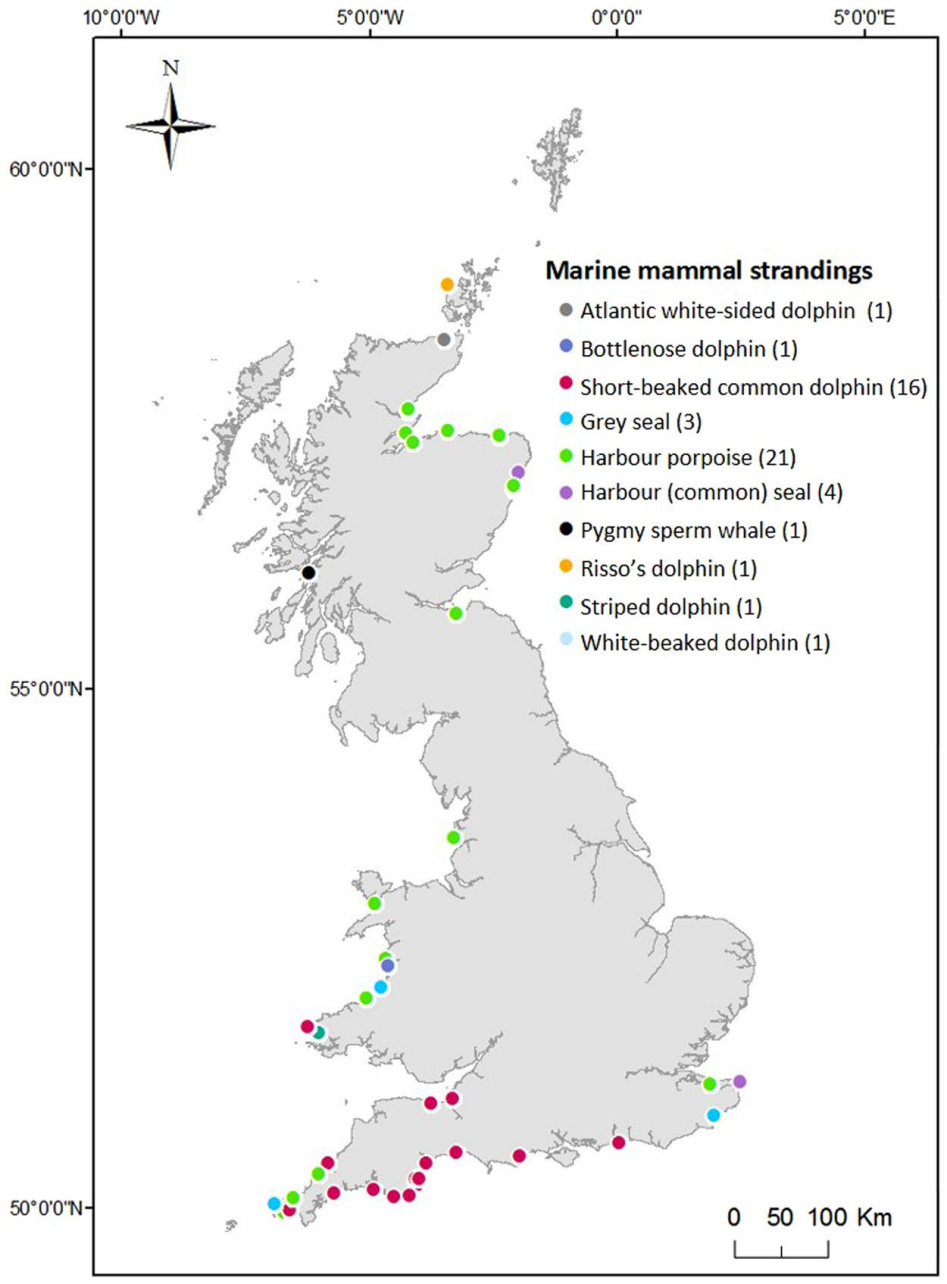
Distribution of marine mammal strandings around the coast of Britain. The coloured points correspond to the marine mammal species and sample size as displayed in the legend. Further details are included in Supplementary Table S5. Map generated using ArcMap 10.3.1.

Post-mortem examinations of 50 stranded marine mammals (Fig. 4, Supplementary Table S5) were carried out by the Scottish Marine Animal Strandings Scheme (SMASS) and the Cetacean Stranding Investigation Programme (CSIP, at the Institute of Zoology and University of Exeter, Penryn campus), during which the gastro-intestinal tracts were extracted and retained for further investigation at Plymouth Marine Laboratory, UK. All post-mortem investigations were conducted using standard procedures^52,53^ by experienced marine mammal pathologists in a necropsy facility rated to biosafety level 2. Samples were collected under contract to Defra and the Devolved Governments of Scotland and Wales. All samples were stored at −20 °C or below.

### Gut content extraction

The GITs were thawed at room temperature before being rinsed with Milli-Q (ultra-pure, filtered) water to remove any unwanted particles (e.g. sand) adhering to the external surfaces. In a clean metal tray, each GIT section – intestines and stomach (stomach compartments for cetaceans) - were cut open separately and the inside rinsed with Milli-Q water. The resulting solution was retained in glass beakers. Due to the relatively low amount of organic material present within some stomach compartments (obvious bony parts and otoliths (ear bones) of fish and squid beaks were picked out), it was possible, using a vacuum pump, to pass the content through 35 µm mesh discs for later inspection. The intestines (and fore-stomachs of some animals) contained a greater amount of material which could obscure microplastic particles upon visual inspection. Therefore, this material was digested using an enzymatic protocol (see below) to remove organic material whilst retaining inorganic and anthropogenic material for inspection (adapted from Lindeque and Smerdon, 2003^54^).

### Enzymatic digestion

Once extracted, the content of the intestines or fore-stomach was placed in a drying oven until the water added during the extraction process evaporated. The dry weight was calculated and the following digestion solution volumes were applied to each 1 g of dried content, the total for each animal varied between 4.5 and 203.5 g. Homogenizing solution (2.2 mL; 400 mM Tris-HCI buffer, 60 mM EDTA, 105 mM NaCl, 1% SDS) was added to the gut content in a clean glass bottle and incubated at 55 °C for 24 hours. A metal spatula was used to physically homogenize the GIT content for 30 seconds, 40 µL of 20 mg mL^−1^ Proteinase K was added and the samples incubated at 55 °C for a further 24 hours. Following this, 400 µL of 5 M sodium perchlorate (NaCLO4) was added and the content physically homogenized for 1 min. Finally, the samples were incubated for 72 hours at 55 °C. Each sample was passed through 35 µm mesh discs (number dependent on amount of material remaining) using a vacuum pump and left to dry at room temperature in a sealed Petri dish.

### Contamination and microplastic loss avoidance

Extensive measures were implemented throughout to limit the risk of contamination of samples by microplastics present on equipment and air-borne particles within in the atmosphere, see below. As a result, no microplastics were found in the procedural blanks and all controls were clear.

### Gut content extraction

For health and safety purposes, nitrile gloves and low-density polyethylene (LDPE) fluid protection gowns were worn over a cotton lab coat. Samples of the gloves and gowns were retained to control for any contamination that may have occurred from these sources. Post-mortem examinations were conducted in an ultra-clean facility and the gut content extraction step was performed inside a positive pressure laminar flow hood with the aim of preventing airborne microplastics from settling on the samples. A damp filter paper in a Petri dish was placed within the hood to catch any such particles, allowing for the efficacy of this measure to be assessed. All equipment was thoroughly rinsed with Milli-Q water and all surfaces were wiped down with 70% ethanol prior to any work commencing. All equipment was rinsed with Milli-Q water again between each GIT section. A procedural blank (50 mL Milli-Q water) was run through the process to control for any contamination at this stage.

### Enzymatic digestion

As above, all equipment was rinsed with Milli-Q and all pipettes and syringes were flushed with Milli-Q prior to use. A procedural blank was run at this stage. Foil lids were used instead of plastic bottle caps as these were previously observed to cause contamination. The metal spatula was rinsed with homogenizing solution (deemed contamination-free after testing) after the homogenising step to avoid loss of particles from samples.

### Vacuum pumping

Prior to filtering, all mesh discs were visually inspected for potential contamination under a microscope and any particles removed. Milli-Q water was run through the vacuum pump and mesh disc to allow for potential contamination from the equipment to be detected and prevented. If particles were found, the vacuum pump and mesh disc were cleaned again until no particles were detected. Only then were samples filtered. The vacuum pump was then flushed copiously with Milli-Q water to ensure no particles became adhered to the edges and so lost from the sample. The vacuum pump was used inside the laminar flow hood to minimise air-borne contamination. Damp filtered paper inside a petri dish was placed alongside the samples to control for any contamination that might have occurred.

### Microplastic identification and characterisation

The mesh discs were visually inspected under an Olympus SZX16 microscope and potential microplastics (identified by colour and uniformity of shape and material; Cole *et al*.^55^; Norén^56^) classified by type (fragment or fibre), colour, size and description, and photographed using a microscope mounted Canon EOS 550D DSLR camera. A sub-sample of one particle from each animal (*n* = 50) was subjected to further analysis using attenuated total reflection-Fourier transform infra-red spectroscopy (ATR-FTIR; PerkinElmer Spotlight 400 FT-IR Imaging System) to confirm the identity of the particles and determine the accuracy level of their visual identification as synthetic materials. Particles were scanned at a resolution of 8 cm^−1^ (wavelength range = 4000–650 cm^−1^) and pixel size of 6.25 µm using *SpectrumIMAGE*^*TM*^ software. The resulting spectra were compared to a spectral database from a number of polymer libraries using *Spectrum*^*TM*^ (PerkinElmer). FTIR was attempted for a greater number of particles (*n* = 65 in total) but obtaining reliable spectra matches was not possible for some due to the extent of degradation. Though these particles were qualitatively similar to those with reliable spectra matches, we were conservative in our inclusion of only particles that exceeded the search score confidence of 0.70 or greater^21^ and those considered to have reliable spectra matches (after visual inspection) as this was deemed the most robust method.

### Factors affecting microplastic abundance

A General Linear Mixed Model (GLMM) was used to examine whether factors such as taxon (cetacean or pinniped), age-class (adult or juvenile), sex (male or female), length of animal and cause-of-death (infectious disease, trauma or other) were related to microplastic abundance. These factors were incorporated within the GLMM as fixed effects and Species was used as a random effect to account for the differing number of animals sampled from each species.

### Distribution of microplastics within GIT

One-way analysis of variance (ANOVA) was used to assess whether microplastic abundance differs between GIT sections in all animals and among stomach compartments (fore, fundic and pyloric) in cetaceans. Statistical significance was set at a probability level (α) of 0.05. Analyses were undertaken in the statistical computing software, R^57^.

## Supplementary information

Supplementary Information

## References

[1] Mössner S, Ballschmiter K (1997). Marine mammals as global pollution indicators for organochlorines. Chemosphere.

[2] Bossart GD (2011). Marine mammals as sentinel species for oceans and human health. Vet. Pathol..

[3] Jepson PD (2016). PCB pollution continues to impact populations of orcas and other dolphins in European waters. Sci. Rep..

[4] Pierce GJ (2008). Bioaccumulation of persistent organic pollutants in female common dolphins (Delphinus delphis) and harbour porpoises (Phocoena phocoena) from western European seas: Geographical trends, causal factors and effects on reproduction and mortality. Environ. Pollut..

[5] Murphy S (2015). Reproductive failure in UK harbour porpoises phocoena phocoena: Legacy of pollutant exposure?. PLoS One.

[6] Parsons ECM (2015). Key research questions of global importance for cetacean conservation. Endanger. Species Res..

[7] Kuhn, S., Bravo Rebolledo, E. L. & van Franeker, J. A. In *Marine Anthropogenic**Litter* (eds Bergmann, M., Gutow, L. & Klages, M.) 75–116 (SpringerLink, 2015).

[8] Lusher AL (2015). Microplastic and macroplastic ingestion by a deep diving, oceanic cetacean: The True’s beaked whale Mesoplodon mirus. Environ. Pollut..

[9] Lusher AL, Hernandez-milian G, Berrow S, Rogan E, Connor IO (2018). Incidence of marine debris in cetaceans stranded and bycaught in Ireland: Recent findings and a review of historical knowledge. Environ. Pollut..

[10] Besseling E (2015). Microplastic in a macro filter feeder: Humpback whale Megaptera novaeangliae. Mar. Pollut. Bull..

[11] Hernandez-Gonzalez A (2018). Microplastics in the stomach contents of common dolphin (Delphinus delphis) stranded on the Galician coasts (NW Spain, 2005–2010). Mar. Pollut. Bull..

[12] Andrady AL (2011). Microplastics in the marine environment. Mar. Pollut. Bull..

[13] Barnes DKA, Galgani F, Thompson RC, Barlaz M (2009). Accumulation and fragmentation of plastic debris in global environments. Philos. Trans. R. Soc. B.

[14] Browne MA (2011). Accumulation of microplastic on shorelines worldwide: Sources and sinks. Environ. Sci. Technol..

[15] Cole M, Lindeque P, Halsband C, Galloway TS (2011). Microplastics as contaminants in the marine environment: A review. Mar. Pollut. Bull..

[16] UNEP. *Marine Litter: A Global Challenge*. *UNEP* (2009).

[17] Boucher, J. & Friot, D. *Primary microplastics in the oceans: a global evaluation of sources*. **43**, 10.2305/IUCN.CH.2017.01.en (2017).

[18] Cole M (2013). Microplastic ingestion by zooplankton. Environ. Sci. Technol..

[19] Farrell P, Nelson K (2013). Trophic level transfer of microplastic: Mytilus edulis (L.) to Carcinus maenas (L.). Environ. Pollut..

[20] Watts AJR (2014). Uptake and retention of microplastics by the shore crab Carcinus maenas. Environ. Sci. Technol..

[21] Lusher AL, McHugh M, Thompson RC (2013). Occurrence of microplastics in the gastrointestinal tract of pelagic and demersal fish from the English Channel. Mar. Pollut. Bull..

[22] Amélineau, F. *et al*. Microplastic pollution in the Greenland Sea: Background levels and selective contamination of planktivorous diving seabirds. *Environ. Pollut*., 10.1016/j.envpol.2016.09.017 (2016). 10.1016/j.envpol.2016.09.017 27616650

[23] Steer, M., Cole, M., Thompson, R. C. & Lindeque, P. K. Microplastic ingestion in fish larvae in the western English Channel. *Environ. Pollut*. 1–10, 10.1016/j.envpol.2017.03.062 (2017). 10.1016/j.envpol.2017.03.062 28408185

[24] Desforges JP, Galbraith M, Ross PS (2015). Ingestion of Microplastics by Zooplankton in the Northeast Pacific Ocean. *Arch Env*. Contam Toxicol.

[25] Hocking DP, Marx FG, Park T, Fitzgerald EMG, Evans AR (2017). A behavioural framework for the evolution of feeding in predatory aquatic mammals. Proc. R. Soc. B.

[26] Nelms, S. E., Galloway, T. S., Godley, B. J., Jarvis, D. S. & Lindeque, P. K. Investigating microplastic trophic transfer in marine top predators. *Environ. Pollut*. 1–9, 10.1016/j.envpol.2018.02.016 (2018). 10.1016/j.envpol.2018.02.016 29477242

[27] Wright SL, Rowe D, Thompson RC, Galloway TS (2013). Microplastic ingestion decreases energy reserves in marine worms. Curr. Biol..

[28] Sussarellu, R. *et al*. Oyster reproduction is affected by exposure to polystyrene microplastics. *Proc. Natl. Acad. Sci*. 201519019, 10.1073/pnas.1519019113 (2016). 10.1073/pnas.1519019113 PMC4780615 26831072

[29] Lei L (2018). Microplastic particles cause intestinal damage and other adverse effects in zebrafish Danio rerio and nematode Caenorhabditis elegans. Sci. Total Environ..

[30] Mattsson K (2017). Brain damage and behavioural disorders in fish induced by plastic nanoparticles delivered through the food chain. Sci. Rep..

[31] Teuten EL (2009). Transport and release of chemicals from plastics to the environment and to wildlife. Philos. Trans. R. Soc. B.

[32] Browne MA, Niven SJ, Galloway TS, Rowland SJ, Thompson RC (2013). Microplastic moves pollutants and additives to worms, reducing functions linked to health and biodiversity. Curr. Biol..

[33] Rochman CM, Hoh E, Kurobe T, Teh SJ (2013). Ingested plastic transfers hazardous chemicals to fish and induces hepatic stress. Sci. Rep..

[34] Neves D, Sobral P, Ferreira JL, Pereira T (2015). Ingestion of microplastics by commercial fish off the Portuguese coast. Mar. Pollut. Bull..

[35] Eriksson C, Burton H (2003). Origins and biological accumulation of small plastic particles in fur seals from Macquarie Island. Ambio.

[36] Lusher AL, O’Donnell C, Officer R, O’Connor I (2016). Microplastic interactions with North Atlantic mesopelagic fish. ICES J. Mar. Sci..

[37] Grellier K, Hammond PS (2006). Robust digestion and passage rate estimates for hard parts of grey seal (Halichoerus grypus) prey. Can. J. Fish. Aquat. Sci..

[38] Mintzer VJ, Gannon DP, Barros NB, Read AJ (2008). Stomach contents of mass-stranded short-finned pilot whales (Globicephala macrorhynchus) from North Carolina. Mar. Mammal Sci..

[39] Levine, G. A., Selberg, K. T., Sweeney, J. C., Stone, L. R. & Campbell, M. Gastric foreign bodies in small odontocetes: A clinical approach. In *IAAAM* 1–5 (2014).

[40] Santana MFM, Moreira FT, Turra A (2017). Trophic transference of microplastics under a low exposure scenario: Insights on the likelihood of particle cascading along marine food-webs..

[41] Hermsen E, Pompe R, Besseling E, Koelmans AA (2017). Detection of low numbers of microplastics in North Sea fish using strict quality assurance criteria. Mar. Pollut. Bull..

[42] Wright SL, Thompson RC, Galloway TS (2013). The physical impacts of microplastics on marine organisms: a review. Environ. Pollut..

[43] Claessens M (2011). Occurrence and distribution of microplastics in marine sediments along the Belgian coast. Mar. Pollut. Bull..

[44] Woodall LC (2014). The deep sea is a major sink for microplastic debris. R. Soc. Open Sci..

[45] Duncan, E. *et al*. Microplastic ingestion ubiquitous in marine turtles. *Glob. Chang. Biol*. 10.1111/gcb.14519 PMC6849705 30513551

[46] Fossi MC (2012). Are baleen whales exposed to the threat of microplastics? A case study of the Mediterranean fin whale (Balaenoptera physalus). Mar. Pollut. Bull..

[47] Desforges, J. *et al*. Effects of polar bear and killer whale derived contaminant cocktails on marine mammal immunity. *Environ. Sci. Technol*. 11431–114339, 10.1021/acs.est.7b03532 (2017). 10.1021/acs.est.7b03532 28876915

[48] Hall AJ (2006). The risk of infection from polychlorinated biphenyl exposure in the harbor porpoise (Phocoena phocoena): A case – control approach. Environ. Health Perspect..

[49] Dris R, Gasperi J, Saad M, Mirande CC, Tassin B (2016). Synthetic fibers in atmospheric fallout: A source of microplastics in the environment?. Mar. Pollut. Bull..

[50] Sebille EV (2015). A global inventory of small fl oating plastic debris. Environ. Res. Lett..

[51] Block BA (2011). Tracking apex marine predator movements in a dynamic ocean. Nature.

[52] Kuiken, T. & Garcia Hartmann, M. Proceedings of the first European Cetacean Society workshop on cetacean pathology: dissection techniques and tissue sampling. in *ECS newsletter* 39 (1991).

[53] Deaville, R. & Jepson, P. D. *UK Cetacean Strandings Investigation Programme Final report for the period 1st January 2005-31st December 2010* (2011).

[54] Lindeque PK, Smerdon GR (2003). Temporal transcription of two Antennapedia class homeobox genes in the marine copepod Calanus helgolandicus. Mar. Biotechnol..

[55] Cole M (2014). Isolation of microplastics in biota-rich seawater samples and marine organisms. Sci. Rep..

[56] Norén, F. *Small plastic particles in coastal Swedish water*s. *KIMO Repor*t (2016).

[57] R Core Team. R: A language and environment for statistical computing. R Foundation for Statistical Computing, Vienna, Austria. at www.R-project.org (2018).

