# Supplementary material for: Microplastics in marine mammals stranded around the British coast: ubiquitous but transitory?
Source: Sci Rep. 2019 Jan 31;9:1075. doi: 10.1038/s41598-018-37428-3 (PMC6355900; doi:10.1038/s41598-018-37428-3)
Supplement: Supplementary file 1 — Supplementary Information [file 41598_2018_37428_MOESM1_ESM.docx]

**Microplastics in marine mammals stranded around the British coast: ubiquitous but transitory?**

Nelms, SE^1, 2+^, Barnett J^3^, Brownlow A^4^, Davison NJ^4^, Deaville R^5^, Galloway TS^6^, Lindeque PK^1^, Santillo D^7^, Godley BJ^2, 3*^

^1^ Plymouth Marine Laboratory, Prospect Place, Plymouth, PL1 3DH, UK

^2^ Centre for Ecology and Conservation, University of Exeter, Cornwall, TR10 9EZ, UK

^3^ Environment and Sustainability Institute, University of Exeter, Cornwall, TR10 9EZ, UK

^4^ Scottish Marine Animal Stranding Scheme, SRUC Veterinary Services, Drummondhill, Inverness, IV2 4JZ, UK

^5^ Cetacean Strandings Investigation Programme, Institute of Zoology, Regent’s Park, London, NW1 4RY, UK

^6^ Biosciences, Geoffrey Pope Building, University of Exeter, Devon, EX4 4QD, UK

^7^ Greenpeace Research Laboratories, Innovation Centre Phase 2, University of Exeter, Devon, EX4 4RN, UK

*^+^Lead author: s.nelms@exeter.ac.uk*

*^*^Corresponding author: b.j.godley@exeter.ac.uk*

**SUPPLEMENTARY INFORMATION**

**Table S1.** Model simplification output from GLMM – All animals

* Lowest AIC score/ most appropriate model

| **Model** | **AIC-score** |
| --- | --- |
| 1: No.MPs ~ Taxon + Age + Sex + Length + Cause + (1\|Species) | 246.3198 |
| 2: No. MPs ~ Age + Sex + Length + Cause + (1\|Species) | 249.7889 |
| 3. No. MPs ~ Sex + Length + Cause + (1\|Species) | 249.4026 |
| 4. No. MPs ~ Length + Cause + (1\|Species) | 248.7458 |
| 5. No. MPs ~ Cause + (1\|Species) | 239.1526* |
| 6. No. MPs ~ (1\|Species) | 246.745 |

**Table S2.** *P*-values for each fixed effect following removal from GLMM (ANOVA) – All animals

* Significant *p*-value (<0.05)

| **Fixed effects** | ***p*-value (α)** |
| --- | --- |
| Taxon | 0.0649 |
| Age | 0.9432 |
| Sex | 0.8730 |
| Length | 0.9545 |
| Cause of death | 0.0114* |

**Table S3**. Model simplification output from GLMM – Harbour porpoise and common dolphin only

* Lowest AIC score/ most appropriate model

| **Model** | **AIC-score** |
| --- | --- |
| 1: No.MPs ~ Age + Sex + Length + Cause + (1\|Species) | 187.0793 |
| 2: No. MPs ~ Sex + Length + Cause + (1\|Species) | 187.0270 |
| 3. No. MPs ~ Length + Cause + (1\|Species) | 186.8954 |
| 4. No. MPs ~ Cause + (1\|Species) | 178.6772* |
| 5. No. MPs ~ (1\|Species) | 187.7520 |

**Table S4.** *P*-values for each fixed effect following removal from GLMM (ANOVA) - Harbour porpoise and common dolphin only

* Significant p-value (<0.05)

| **Fixed effects** | **p-value (α)** |
| --- | --- |
| Age | 0.8797 |
| Sex | 0.5473 |
| Length | 0.3994 |
| Cause of death | 0.0076* |

**Table S5.** Life-history information for stranded marine mammals and the associated number of microplastics detected

| **Sample ID** | **Year found** | **Species** | **Taxa** | **Age** | **Sex** | **Length** | **Location** | **Cause of death** | **Total no. MPs** |
| --- | --- | --- | --- | --- | --- | --- | --- | --- | --- |
| EX\|C17\|16 | 2016 | Common dolphin | Cetacean | Juvenile | Male | 192 | South-west England | Trauma | 4 |
| EX\|C18\|16 | 2016 | Harbour porpoise | Cetacean | Juvenile | Female | 120 | South-west England | Trauma | 5 |
| EX\|C19\|16 | 2016 | Harbour porpoise | Cetacean | Juvenile | Male | 113 | South-west England | Trauma | 6 |
| EX\|C20\|16 | 2016 | Harbour porpoise | Cetacean | Juvenile | Female | 115.5 | South-west England | Other | 2 |
| EX\|C21\|16 | 2016 | Common dolphin | Cetacean | Adult | Male | 214 | South-west England | Trauma | 1 |
| EX\|C24\|16 | 2016 | Common dolphin | Cetacean | Juvenile | Male | 162 | South-west England | Other | 1 |
| EX\|C28\|16 | 2016 | Common dolphin | Cetacean | Adult | Female | 204 | South-west England | Other | 5 |
| EX\|S9\|16 | 2016 | Grey seal | Pinniped | Juvenile | Female | 90 | South-west England | Infectious disease | 8 |
| M102/16 | 2016 | Harbour porpoise | Cetacean | Juvenile | Male | 104 | Scotland | Infectious disease | 8 |
| M104/16 | 2016 | Harbour porpoise | Cetacean | Juvenile | Female | 111 | Scotland | Trauma | 4 |
| M109/16 | 2016 | Harbour porpoise | Cetacean | Juvenile | Male | 118 | Scotland | Trauma | 7 |
| M126/16 | 2016 | Harbour porpoise | Cetacean | Juvenile | Female | 129 | Scotland | Trauma | 2 |
| M134/16 | 2016 | Harbour porpoise | Cetacean | Juvenile | Male | 118 | Scotland | Other | 3 |
| M150/16 | 2016 | Harbour porpoise | Cetacean | Adult | Female | 160 | Scotland | Other | 7 |
| M157/16 | 2016 | Risso's dolphin | Cetacean | Juvenile | Male | 254 | Scotland | Infectious disease | 9 |
| M178/16 | 2016 | Harbour porpoise | Cetacean | Juvenile | Male | 111 | Scotland | Trauma | 4 |
| M190/16 | 2016 | Harbour porpoise | Cetacean | Juvenile | Male | 125 | Scotland | Trauma | 5 |
| M191/16 | 2016 | Harbour porpoise | Cetacean | Adult | Female | 154 | Scotland | Trauma | 6 |
| M256/11 | 2011 | Pygmy sperm whale | Cetacean | Adult | Male | 211 | Scotland | Other | 4 |
| M267/16 | 2016 | White-beaked dolphin | Cetacean | Adult | Male | 264 | Scotland | Infectious disease | 3 |
| M273/16 | 2016 | Harbour porpoise | Cetacean | Adult | Male | 143 | Scotland | Infectious disease | 11 |
| M299/16 | 2016 | Atlantic white-sided dolphin | Cetacean | Adult | Male | 246 | Scotland | Other | 8 |
| M444/14 | 2014 | Harbour seal (Common seal) | Pinniped | Juvenile | Female | 98 | Scotland | Infectious disease | 5 |
| M54/16 | 2016 | Harbour seal (Common seal) | Pinniped | Juvenile | Male | 118 | Scotland | Trauma | 4 |
| SS2015/316 | 2015 | Harbour seal (Common seal) | Pinniped | Adult | Male | 172 | East England | Infectious disease | 7 |
| SS2015/317 | 2015 | Harbour seal (Common seal) | Pinniped | Juvenile | Female | 126 | East England | Other | 1 |
| SS2016/301 | 2016 | Grey seal | Pinniped | Juvenile | Male | 150 | East England | Infectious disease | 4 |
| SS2017/6 | 2017 | Grey seal | Pinniped | Juvenile | Female | 123 | West Wales | Infectious disease | 6 |
| SW2015/341 | 2015 | Harbour porpoise | Cetacean | Juvenile | Female | 104 | West Wales | Trauma | 7 |
| SW2015/422 | 2015 | Striped dolphin | Cetacean | Juvenile | Male | 180 | West Wales | Other | 7 |
| SW2016/210 | 2016 | Harbour porpoise | Cetacean | Adult | Female | 157 | West Wales | Trauma | 4 |
| SW2016/280 | 2016 | Harbour porpoise | Cetacean | Juvenile | Female | 122 | West Wales | Trauma | 2 |
| SW2016/317 | 2016 | Harbour porpoise | Cetacean | Adult | Male | 137 | West England | Other | 4 |
| SW2016/397 | 2016 | Harbour porpoise | Cetacean | Adult | Female | 152 | West Wales | Infectious disease | 10 |
| SW2016/402 | 2016 | Bottlenose dolphin | Cetacean | Juvenile | Male | 145 | West Wales | Trauma | 6 |
| SW2016/411 | 2016 | Common dolphin | Cetacean | Adult | Male | 220 | South-west England | Other | 2 |
| SW2016/416 | 2016 | Common dolphin | Cetacean | Juvenile | Female | 165 | South-west England | Infectious disease | 8 |
| SW2016/446 | 2016 | Common dolphin | Cetacean | Adult | Female | 194 | South-west England | Other | 4 |
| SW2016/447 | 2016 | Common dolphin | Cetacean | Adult | Female | 202 | South-west England | Infectious disease | 7 |
| SW2016/477 | 2016 | Common dolphin | Cetacean | Juvenile | Male | 189 | South-west England | Infectious disease | 3 |
| SW2016/478 | 2016 | Common dolphin | Cetacean | Adult | Male | 207 | South-west England | Infectious disease | 4 |
| SW2016/520 | 2016 | Harbour porpoise | Cetacean | Adult | Male | 138 | East England | Other | 5 |
| SW2016/562 | 2016 | Common dolphin | Cetacean | Adult | Male | 225 | West Wales | Infectious disease | 12 |
| SW2017/12 | 2017 | Common dolphin | Cetacean | Juvenile | Female | 170 | South-west England | Infectious disease | 7 |
| SW2017/13 | 2017 | Harbour porpoise | Cetacean | Adult | Male | 140 | South-west England | Trauma | 2 |
| SW2017/15 | 2017 | Common dolphin | Cetacean | Juvenile | Male | 158 | South-west England | Other | 11 |
| SW2017/2 | 2017 | Harbour porpoise | Cetacean | Juvenile | Male | 117 | South-west England | Trauma | 6 |
| SW2017/60 | 2017 | Common dolphin | Cetacean | Juvenile | Female | 177 | South-west England | Other | 9 |
| SW2017/77 | 2017 | Common dolphin | Cetacean | Juvenile | Female | 180 | South-west England | Other | 8 |
| SW2017/8 | 2017 | Common dolphin | Cetacean | Adult | Male | 194 | South-west England | Trauma | 5 |
